# Exploring the Potential of an AI Chatbot as a Supplementary Tool for Nutritional Prescription Hospital Discharge: A Preliminary Study

**DOI:** 10.1155/sci5/2632410

**Published:** 2025-10-26

**Authors:** Renato Augusto da Cruz Pereira, Raianne Rodrigues Lima, Amanda Cristina Araujo Gomes, Fernanda Araújo Santos Saldanha, Dino Schwingel, Paulo Adriano Schwingel, Bruno Bavaresco Gambassi

**Affiliations:** ^1^Post-Graduation Program on Management and Health Programs and Services (PPGGPSS), CEUMA University (UNICEUMA), São Luís MA 65075-120, Brazil; ^2^University Hospital of the Federal University of Maranhão (HU-UFMA), Brazilian Hospital Services Company (EBSERH), São Luís MA 65020-070, Brazil; ^3^Human Performance Research Laboratory (LAPEDH), University of Pernambuco (UPE), Petrolina PE 56328-900, Brazil; ^4^AI-Assisted Diagnostics Research Group (AIDRG), University of Pernambuco (UPE), Petrolina PE 56328-900, Brazil

## Abstract

AI-based chatbots are increasingly used to automate clinical documentation, but their efficacy in generating specialized nutritional prescriptions for hospital discharge remains underexplored. This preliminary study evaluated the performance of a prominent AI chatbot in producing clinically valid nutritional guidelines. A specialist committee of registered dietitians selected 16 common medical and surgical pathologies. Standardized prompts were used to generate nutritional discharge guidelines from the chatbot. The same committee then evaluated the AI-generated texts for technical accuracy and content presentation on a 0–10 scale (approval score ≥ 7.0). Inter-rater reliability was assessed using the intraclass correlation coefficient (ICC) and Cohen's Kappa. Overall, 50% (8/16) of the AI-generated prescriptions met the predefined approval threshold. Performance was higher for medical pathologies (mean score: 7.1 ± 1.2) compared to surgical pathologies (6.6 ± 1.4), although this difference was not statistically significant (*p* > 0.05). Inter-rater reliability was substantial (ICC > 0.72; Kappa > 0.62). The findings indicate that AI chatbots hold promise as supplementary tools for drafting nutritional discharge summaries, potentially reducing administrative workload. However, their variable performance underscores the indispensable need for rigorous review and validation by qualified healthcare professionals before any clinical application.

## 1. Introduction

Artificial intelligence (AI) encompasses technologies designed to replicate the sophisticated cognitive functions traditionally associated with human intellect. A notable subset of these technologies is large language models (LLMs), which are trained on extensive datasets of textual data. This training allows LLMs to generate responses that closely mimic human communication in terms of complexity, context awareness, and nuance. Prominent examples of these advanced conversational agents include ChatGPT (OpenAI, L.L.C., San Francisco, CA, USA) and Gemini, formerly known as Bard (Google LLC, Mountain View, CA, USA) [1–4].

The integration of AI technologies into healthcare has catalyzed significant transformations by improving clinical documentation, supporting decision-making processes, and personalizing patient care [1, 5, 6]. This includes promising applications such as predicting potential medication errors and improving diagnostic accuracy. However, this potential is tempered by significant challenges. A primary concern is the risk of generating factually incorrect or fabricated information—a phenomenon known as “artificial hallucination” [7]—and the absence of clear regulatory frameworks. As noted in a comprehensive systematic review by Sallam [8], the utility of these tools is marked by both “promising perspectives and valid concerns,” underscoring that their integration requires a cautious, evidence-based approach [9].

In this context, hospital discharge planning is a critical component of patient care continuity that demands precision and comprehensiveness. Inadequacies in this process are associated with compromised patient outcomes and increased readmission rates [10, 11]. Efficient, standardized discharge procedures, particularly those related to nutritional counseling, are essential for promoting patient adherence to prescribed care regimens after discharge [10, 12].

Although generative AI models are useful for general medical documentation [11–13], their use in generating structured nutritional discharge instructions has not been widely explored. Recent evidence suggests that AI chatbots can support healthcare professionals by automating routine documentation tasks [1, 14, 15]. However, their reliability and accuracy vary substantially with the complexity of the clinical scenario. For example, studies have shown that nutritional recommendations generated by chatbots can have significant caloric discrepancies, include foods that are inappropriate for specific pathologies, and lack personalization for patients with multiple comorbidities [16–24]. Furthermore, persistent concerns regarding the accuracy, completeness, and reproducibility of the advice generated by these tools underscore the necessity of validation by trained healthcare professionals [25, 26].

While a growing body of literature evaluates the performance of AI chatbots in answering general nutritional questions and their accuracy on standardized medical exams, a critical gap remains [23, 27]. To our knowledge, no study has systematically evaluated the specific utility of these models for generating structured, patient-ready nutritional discharge summaries—a key component of care transition. Addressing this gap, the present preliminary study explores the effectiveness of Google's AI chatbot in generating nutritional discharge guidelines. This investigation aims not only to assess the chatbot's capability in providing reliable, structured, and contextually accurate nutritional guidance but also to examine its integration into hospital discharge workflows. The study utilizes detailed clinical scenarios and evaluations conducted by dietitians to assess the clinical validity and applicability of AI-generated nutritional prescriptions. By doing so, this research contributes novel insights into how generative AI can potentially standardize and automate nutritional documentation, addressing crucial methodological gaps through standardized reporting methods [28, 29].

Importantly, this study underscores that despite technological advancements, AI tools remain adjunctive rather than replacements for professional clinical judgment. Ethical considerations, patient safety, and accountability must guide the integration of these technologies into clinical workflows. Thus, this research also briefly reflects on the ethical implications and practical limitations inherent in deploying generative AI tools in healthcare settings [26, 30, 31].

By addressing the highlighted methodological and conceptual shortcomings identified in prior literature and by reviewers, the current study significantly advances the understanding of generative AI applications in clinical nutrition, thereby laying the foundation for future research and practical integration of these promising technologies in healthcare systems.

## 2. Materials and Methods

**Figure 1:**
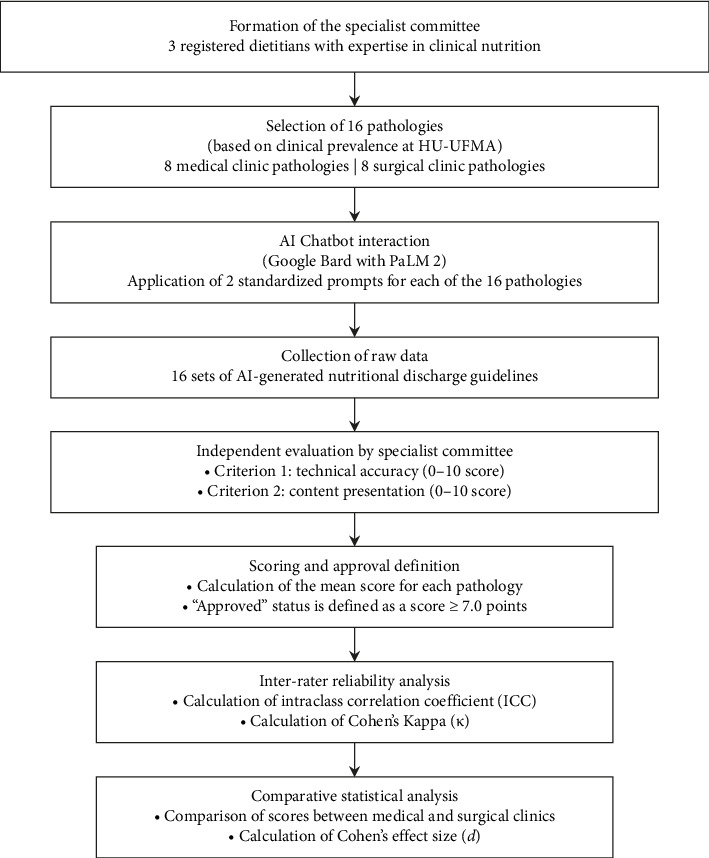
Flowchart of the study design.

This preliminary efficacy study was designed and is reported following the Strengthening the Reporting of Observational Studies in Epidemiology (STROBE) guidelines to ensure transparency and methodological rigor [28, 29]. The study was designed to evaluate the capabilities of Google's AI chatbot in producing clinically accurate and contextually appropriate nutritional discharge guidelines. The research was systematically structured into three sequential phases to ensure a comprehensive evaluation (Figure 1).

First, a Committee of Specialists was formed, composed of three registered dietitians with specialist titles in Clinical Nutrition and Hospital Nutrition Residency [20, 21, 24]. The committee was responsible for selecting 16 pathologies frequently encountered in clinical practice at the University Hospital of the Federal University of Maranhão (HU-UFMA). Specifically, the committee identified the eight most prevalent medical conditions and the eight most prevalent surgical conditions requiring nutritional management at discharge, providing a balanced representation of clinical scenarios (Table 1).

In the second phase, which took place in December 2023, Google's AI chatbot (then named Google Bard1) was chosen as the generative AI tool for several technical and operational reasons. The hospital's stringent data security policies required an AI platform that could be seamlessly integrated via an Application Programming Interface (API) and align effectively with the existing IT infrastructure. Although other chatbots like ChatGPT (OpenAI, L.L.C.) and Bing (Microsoft Corporation, Redmond, WA, USA) demonstrated advanced reasoning capabilities, practical considerations such as API accessibility, continuous data updating, and compatibility with existing infrastructure made Google's AI chatbot the most suitable option.

In the subsequent data collection phase, standardized prompts were meticulously designed and consistently applied across all 16 pathologies to ensure uniformity and reproducibility. Two sequential prompts were designed for each pathology to minimize memory biases [14, 32]. The initial prompt was consistently structured as follows in the Brazilian Portuguese language: “*Gostaria que você me auxiliasse a construir um texto de alta hospitalar com orientações nutricionais que devem ser seguidas pelo paciente em domicílio. Imagine que você é alguém com um conhecimento muito amplo sobre Nutrição e a patologia selecionada. Dê orientações gerais, exemplos de alimentos que podem ser consumidos e os que devem ser evitados. Não dê sugestões específicas sobre medicações ou tratamentos médicos ou de outras categorias profissionais que não seja a nutrição*.” [“Please assist in drafting a hospital discharge summary with nutritional guidelines for the patient to follow at home. Act as an expert with extensive knowledge in both Nutrition and the relevant pathology. Provide general dietary recommendations, including examples of foods that should be consumed and those that should be avoided. Do not include any suggestions related to medications, medical treatments, or guidance beyond the professional scope of nutrition.”]

A subsequent prompt, also in the Brazilian Portuguese language, requested explicit justification and evidence sources: “*Revise o seu texto e justifique a sua resposta apresentando as fontes que embasaram você na construção dessa orientação*.” [“Review your previous response. Then, justify the guidelines you provided by citing the sources that were used to construct them.”] This standardization allowed for a comprehensive evaluation of the outputs for each clinical scenario. Complete interactions were documented and are available via links in Table 2.

The Specialist Committee evaluated the responses in detail using two primary criteria: technical accuracy and content presentation [20, 21, 24]. Technical accuracy encompassed four subcategories, each worth up to 2.5 points: (1) scientific foundation and evidence-based approach, (2) safety and adequacy of the profile, (3) completeness and specificity of the prescription, and (4) absence of factual errors or harmful recommendations. Content presentation also included four subcategories (2.5 points each): (1) clarity, objectivity, and accessible language; (2) logical organization and content structure; (3) tone, empathy, and educational value; and (4) practicality and actionability of recommendations.

The evaluation results were quantified using a scoring scale from 0.0 to 10.0, requiring a minimum score of 7.0 for approval. This threshold was deliberately selected based on established practices in health profession education and reflects a critical minimum competency level for safe clinical practice. The 7.0 standard corresponds to rigorous benchmarks commonly used in health profession education and embodies critical competencies for safe clinical practice, risk management, and psychometric robustness, which are commonly applied in medical and nutrition residency programs [33–36].

To confirm the consistency of the evaluations among the three specialist reviewers, statistical reliability analyses were conducted. These analyses revealed strong inter-rater agreement: the intraclass correlation coefficient (ICC) was 0.726 for technical accuracy and 0.818 for content presentation [32, 37]. Furthermore, Cohen's Kappa, which evaluates the agreement between “Approved” and “Not Approved,” yielded values of 0.625 and 0.766 for technical accuracy and content presentation, respectively, indicating substantial reliability [38].

Data management and statistical analyses were performed using the Statistical Package for the Social Sciences (SPSS) for Windows (SPSS Inc., Chicago, IL, USA, release 16.0.2, 2008). A robust double-entry method was applied to validate the consistency and accuracy of data entry. Descriptive statistics were utilized to delineate categorical variables as absolute and relative frequencies, and continuous variables were presented as means ± standard deviations. The normality of data distribution was verified using the Shapiro–Wilk test. In a comparative analysis between responses related to medical and surgical clinics [9], independent samples *t*-tests were employed for continuous variables, while Fisher's exact test was used for categorical variables. Additionally, the magnitude of differences observed during the evaluation phases was quantified using Cohen's *d*, an effect size measure. All statistical analyses adhered to a two-tailed approach, with exact *p* values and 95% confidence intervals (CI_95%_) provided. The threshold for statistical significance was established at a *p* value of ≤ 0.05.

Since the research involved only AI chatbot interactions, ethical approval was not necessary.

## 3. Results

The evaluation of nutritional prescriptions generated by Google's AI chatbot revealed varied performance across the 16 selected pathologies. Among these, eight (50.0%) achieved the minimum approval score of 7.0 or higher and were classified as “Approved.” These approved pathologies were systemic arterial hypertension, hepatic cirrhosis, coronary artery disease, diabetes mellitus, hip arthroplasty, laparoscopic cholecystectomy, myocardial revascularization, and rectosigmoidectomy, as detailed in Table 3.

**Figure 2:**
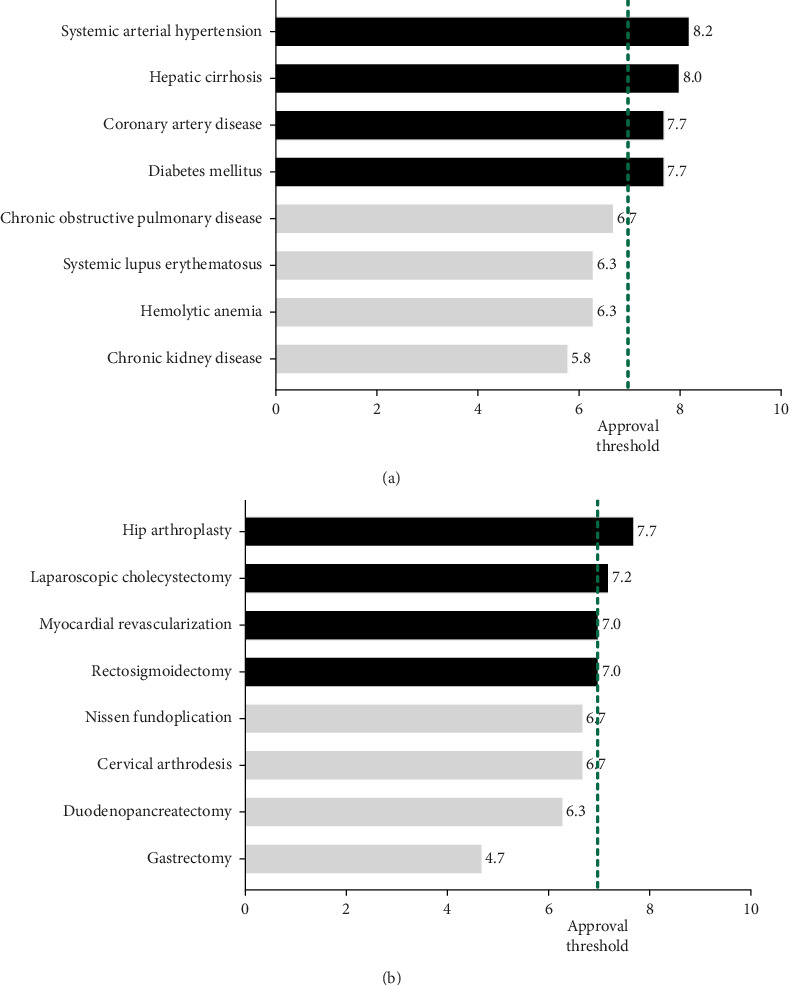
Mean performance scores of AI-generated nutritional hospital discharge guidelines, stratified by clinical area. (a) Eight common medical conditions and (b) eight common surgical conditions. Bars denote the mean score; the dashed vertical line marks the minimum approval threshold (score ≥ 7.0) for clinical acceptability.

Notably, the approved pathologies were evenly distributed between the medical and surgical clinics (four from each). However, when analyzed by clinical setting, only the medical clinic, as a group, reached the approval criterion with a mean (SD) score of 7.1 (±1.2) points, while the surgical clinic yielded a slightly lower aggregate score of 6.6 (±1.4) points, which did not meet the approval threshold (Figure 2).

A comparative statistical analysis between the two clinics revealed no statistically significant differences for either technical accuracy (*p*=0.21) or content presentation (*p*=0.33). Cohen's *d* effect size calculation corroborated this finding, revealing only a small effect size between the clinics for both technical accuracy (*d* = 0.37, CI_95%_: −0.20–0.94) and content presentation (*d* = 0.29, CI_95%_: −0.29–0.85) (Table 4).

## 4. Discussion

This study provides compelling preliminary evidence that a generative AI chatbot can serve as a valuable supplementary tool in producing structured and technically sound nutritional discharge prescriptions. Our findings revealed that half of the AI-generated guidelines for 16 distinct clinical pathologies met a predefined clinical competency threshold. This highlights both the significant potential of these tools in automating routine clinical documentation and the critical need for domain-specific validation, as performance was notably more effective for common medical conditions compared to complex surgical scenarios.

The disparity in performance observed, in which pathologies such as systemic arterial hypertension and diabetes mellitus received higher scores than complex surgical cases such as gastrectomy and duodenopancreatectomy, warrants careful consideration. This variance likely reflects the underlying training data of the LLM. Medical conditions with high prevalence are extensively documented in publicly accessible medical literature, scientific papers, and clinical guidelines, forming a rich corpus for AI models [22, 23, 30]. This discrepancy may also be explained by the fundamental difference between declarative and procedural knowledge. Nutritional management for chronic medical conditions often relies on declarative knowledge, which is a stable set of facts and guidelines. In contrast, postsurgical nutritional care is highly procedural, involving dynamic, phase-based protocols (e.g., transitioning from clear liquids to solid foods) that depend heavily on context, such as patient tolerance and recovery milestones. Current LLMs excel at retrieving and structuring declarative information, but they often struggle with the multistep, context-dependent reasoning required for procedural tasks [39, 40]. This architectural limitation likely contributes to lower performance in complex surgical scenarios, which demand more than factual recall. Our exploratory bibliometric search on PubMed® supports this hypothesis, revealing a substantially greater volume of publications for the selected medical conditions. This suggests a data availability bias that influences the model's proficiency.

Our results align with a growing body of research that positions AI chatbots as powerful assistants for streamlining clinical workflows. The potential to reduce the administrative burden of creating discharge summaries, as observed in our study, resonates with the conclusions of Patel and Lam [26] and Singh et al. [21]. However, mirroring their findings, our work also underscores that this automation cannot replace professional judgment. The 50% approval rate in our study is comparable to the ∼58% of “correct” or “almost completely correct” responses found by Johnson et al. [19], reinforcing the notion that while promising, these tools are not yet infallible. This necessity for a “human-in-the-loop” is a central theme in the responsible implementation of AI in healthcare, consistent with findings that high-quality automated medical summarization remains unfeasible without expert postediting. This professional oversight is crucial not only for verifying factual accuracy but also for incorporating the uniquely human skills of clinical practice: interpreting nonverbal cues during counseling, addressing psychosocial and economic barriers to adherence, and adapting recommendations based on a patient's real-world context and emotional state—elements that current AI models cannot assess [30, 31].

It is important to note that while the 7.0 approval threshold provided a necessary quantitative benchmark for our analysis, the practical distinction between scores just below this cutoff (e.g., 6.7) and those achieving it may be subtle. These “borderline” cases do not indicate a total failure of the AI. Rather, they demonstrate the AI's significant potential to produce a functionally useful initial draft. At the same time, they underscore the indispensable need for a qualified professional to conduct a meticulous review and refinement to ensure that the final recommendations meet the standards of clinical safety and personalization. Therefore, these instances strongly reinforce our central conclusion: AI chatbots should be used as sophisticated drafting assistants to accelerate clinical documentation rather than as autonomous substitutes for professional clinical judgment.

This study has several notable strengths, including its novel focus on nutritional discharge summaries, a critical but under-researched application of AI. The use of a structured evaluation framework, assessed by a committee of clinical nutrition specialists, and the robust inter-rater reliability (ICC and Cohen's Kappa) ensure the validity of our findings. Furthermore, while the comparison between medical and surgical settings did not yield statistically significant differences, the calculated small-to-medium effect size (Cohen's *d* = 0.37 for technical accuracy) suggests a potential underlying trend that our study may have been underpowered to detect. Future research with larger sample sizes would be valuable to confirm if a true difference exists. Nonetheless, certain limitations must be acknowledged. First, this is a preliminary study involving a single AI chatbot whose performance represents a “snapshot in time” of a rapidly evolving technology. The update from Bard to the more advanced Gemini model shortly after our data collection highlights this challenge, indicating that the current performance may already differ from our findings. This highlights a fundamental limitation for all research in this field [9]. Second, our evaluation was based on predefined clinical scenarios and did not involve a direct comparison with discharge summaries written by human dietitians, which would represent a “gold standard.” Third, our findings are based on a single AI platform and a selection of pathologies based on prevalence at one university hospital. The performance of AI models can vary significantly when applied to different clinical settings or patient populations. This challenge is known as a lack of generalizability or transportability. Therefore, external validation of these findings in multicenter studies using diverse AI platforms is critical before broader implementation can be considered [41–43]. This aligns with broader critical questions in AI research that emphasize interpreting findings from single-center evaluations with caution until they are replicated across various contexts [44].

Furthermore, clearly defining guidelines regarding accountability, data privacy, and the management of potential AI-generated errors is essential for the ethical integration of these tools into clinical practice [9, 30]. In the Brazilian context, the use of external AI chatbots must align with the Brazilian General Data Protection Law (LGPD), raising critical questions about how third-party technology companies transmit, store, and process patient data in prompts [45, 46]. Equally important are the unresolved questions of clinical accountability. In the event of patient harm resulting from erroneous AI-generated advice, determining legal liability—whether it rests with the supervising clinician, the implementing institution, or the AI developer—remains a complex and largely untested legal issue [47]. These challenges underscore the necessity of maintaining a “human-in-the-loop” approach to ensure patient safety and professional accountability.

The primary clinical implication of our findings is that AI chatbots, in their current state, should be regarded as sophisticated drafting tools, not autonomous prescribers. They can accelerate the creation of a baseline discharge summary, which must then be critically reviewed, edited, and personalized by a qualified dietitian. This approach leverages AI's efficiency while safeguarding patient safety through essential human expertise. Furthermore, the ethical integration of these tools into clinical practice requires clear guidelines regarding accountability, data privacy, and the management of potential AI-generated errors [7, 28]. Future research should focus on several key areas: (1) conducting comparative studies across multiple LLMs (e.g., Gemini, GPT-4, and Claude); (2) validating AI-generated content against human-created, gold-standard documents in real-world clinical trials; and (3) exploring methods to improve AI accuracy through fine-tuning with specialized, high-quality clinical datasets, particularly for surgical and other complex nutritional domains.

In conclusion, this study contributes to the growing body of evidence supporting the supplementary use of AI in healthcare. By demonstrating both the potential and the current limitations of an LLM for generating nutritional discharge guidelines, our work reinforces the need for a cautious, evidence-based approach to integrating these powerful technologies. The path forward lies not in replacing clinicians but in empowering them with validated, reliable, and ethically governed AI tools that enhance the quality and efficiency of patient care.

## 5. Conclusions

This preliminary study demonstrated that Google's AI chatbot can generate clinically acceptable nutritional discharge guidelines, particularly for common medical pathologies. Our findings support its use as a supplementary tool to streamline the creation of initial drafts, thereby reducing the administrative burden on healthcare professionals. However, the observed variability in performance, especially in complex surgical scenarios, underscores that all AI-generated content requires rigorous validation and personalization by a qualified dietitian to ensure patient safety and clinical appropriateness. Future advancements in AI, coupled with targeted training on specialized clinical data, will be essential to fully realize the potential of these technologies to enhance efficiency and effectiveness in healthcare.

## Figures and Tables

**Table 1:** Pathologies selected for AI evaluation based on clinical prevalence at the University Hospital of the Federal University of Maranhão (HU-UFMA) from Brazilian Hospital Services Company (EBSERH).

Medical clinic	Surgical clinic
Chronic kidney disease	Cervical arthrodesis
Chronic obstructive pulmonary disease	Duodenopancreatectomy
Coronary artery disease	Gastrectomy
Diabetes mellitus	Hip arthroplasty
Hemolytic anemia	Laparoscopic cholecystectomy
Hepatic cirrhosis	Myocardial revascularization
Systemic arterial hypertension	Nissen fundoplication
Systemic lupus erythematosus	Rectosigmoidectomy

**Table 2:** Selected pathologies and links for prompt access.

Pathologies	Link to access the AI chatbot conversation
Cervical arthrodesis	https://g.co/gemini/share/09ace7f0cf95
Chronic kidney disease	https://g.co/gemini/share/693290329298
Chronic obstructive pulmonary disease	https://g.co/gemini/share/3aff4288e095
Coronary artery disease	https://g.co/gemini/share/c1bbe1f735e3
Diabetes mellitus	https://g.co/gemini/share/df01edcb64b9
Duodenopancreatectomy	https://g.co/gemini/share/b1500696ef89
Gastrectomy	https://g.co/gemini/share/4ed62e0b61e7
Hip arthroplasty	https://g.co/gemini/share/2635862d77b5
Hemolytic anemia	https://g.co/gemini/share/ee430669c586
Hepatic cirrhosis	https://g.co/gemini/share/2f00030ea0cd
Laparoscopic cholecystectomy	https://g.co/gemini/share/7ce86b37fdef
Myocardial revascularization	https://g.co/gemini/share/debcbd414dad
Nissen fundoplication	https://g.co/gemini/share/d7f2d8d5a9a7
Rectosigmoidectomy	https://g.co/gemini/share/306157bae5fe
Systemic arterial hypertension	https://g.co/gemini/share/4d526808b3e2
Systemic lupus erythematosus	https://g.co/gemini/share/3d61e4d1503a

**Table 3:** Performance scores and approval status of artificial intelligence (AI)–generated nutritional prescriptions by pathology.

Pathologies	Technical accuracy	Content presentation	Average score	Approval status
	(Mean ± SD)	(Mean ± SD)		
Medical clinic	7.0 ± 1.4	7.2 ± 1.0	7.1	Approved
Systemic arterial hypertension	8.7 ± 1.2	7.7 ± 0.6	8.2	Approved
Hepatic cirrhosis	7.7 ± 0.6	8.3 ± 0.6	8.0	Approved
Coronary artery disease	7.7 ± 0.6	7.7 ± 0.6	7.7	Approved
Diabetes mellitus	7.7 ± 0.6	7.7 ± 0.6	7.7	Approved
Chronic obstructive pulmonary disease	6.7 ± 1.5	6.7 ± 1.5	6.7	Not approved
Systemic lupus erythematosus	6.3 ± 0.6	6.3 ± 0.6	6.3	Not approved
Hemolytic anemia	6.0 ± 1.7	6.7 ± 1.5	6.3	Not approved
Chronic kidney disease	5.3 ± 0.6	6.3 ± 0.6	5.8	Not approved
Surgical clinic	6.5 ± 1.6	6.8 ± 1.3	6.6	Not approved
Hip arthroplasty	7.7 ± 0.6	7.7 ± 0.6	7.7	Approved
Laparoscopic cholecystectomy	6.7 ± 1.5	7.7 ± 0.6	7.2	Approved
Myocardial revascularization	7.0 ± 1.0	7.0 ± 1.0	7.0	Approved
Rectosigmoidectomy	7.0 ± 1.0	7.0 ± 1.0	7.0	Approved
Nissen fundoplication	6.3 ± 1.5	7.0 ± 2.6	6.7	Not approved
Cervical arthrodesis	6.7 ± 1.5	6.7 ± 1.5	6.7	Not approved
Duodenopancreatectomy	6.3 ± 0.6	6.3 ± 0.6	6.3	Not approved
Gastrectomy	4.0 ± 2.6	5.3 ± 0.6	4.7	Not approved

Abbreviation: SD, standard deviation.

**Table 4:** Statistical comparison of artificial intelligence (AI) performance between medical and surgical clinics.

Criteria	Medical clinic			Surgical clinic			*p*	*d* (95% CI)
	Mean	±	SD	Mean	±	SD		
Technical accuracy	7.0	±	1.4	6.5	±	1.6	0.209	0.37 (−0.20–0.94)
Content presentation	7.2	±	1.0	6.8	±	1.3	0.328	0.29 (−0.29–0.85)

*Note: d*, Cohen's *d* effect size.

Abbreviations: CI, confidence interval; SD, standard deviation.

## Data Availability

The data used to support the findings of this study are included within the article.

## Endnotes

^1^Google Bard was renamed Google Gemini in February 2024.
